# Supplementary material for: Collagen Network Formation in In Vitro Models of Musculocontractural Ehlers–Danlos Syndrome
Source: Genes (Basel). 2023 Jan 24;14(2):308. doi: 10.3390/genes14020308 (PMC9957042; doi:10.3390/genes14020308)
Supplement: Supplementary file 1 [file genes-14-00308-s001.zip › genes-2098819-supplementary material.pdf]

Supplemental Table S1. Disaccharide composition of CS and DS in the conditioned medium of skin fibroblast cultures from an mcEDS-*CHST14* patient

The conditioned medium of skin fibroblast cultures derived from an mcEDS-*CHST14* patient was individually digested with chondroitinase AC or B for the specific analysis of CS and DS moiety, respectively. Each digest was labeled with 2AB, and analyzed by anion-exchange HPLC (Supplemental Figure S1). The amount of resultant disaccharides in each sample was calculated based on the peak area in each chromatogram.

	nmol/mg protein	
	**CS**	**DS**
ΔHexA-GalNAc	N.D.[a]	N.D.
ΔHexA-GalNAc(6S)	31.2	N.D.
ΔHexA-GalNAc(4S)	12.8	N.D.
ΔHexA(2S)-GalNAc(6S)	N.D.	N.D.
ΔHexA(2S)-GalNAc(4S)	N.D.	N.D.
ΔHexA-GalNAc(4S,6S)	N.D.	N.D.
ΔHexA(2S)-GalNAc(4S,6S)	N.D.	N.D.
Total CS disaccharide	44.0	N.D.

a, not detected (<0.01 nmol/mg).

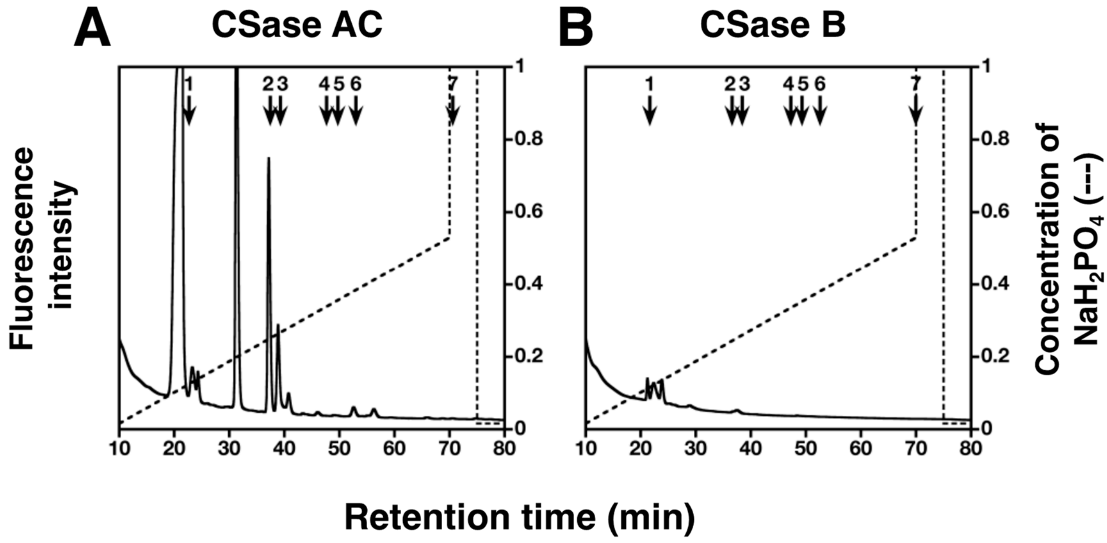

Supplemental Figure S1. HPLC profiles of the digests of the conditioned medium from skin fibroblasts cultures with chondroitinase AC or B.

The conditioned medium of skin fibroblast cultures derived from an mcEDS-*CHST14* patient was individually digested with chondroitinase AC (*A*) or B (*B*) for the specific analysis of CS or DS moiety, respectively. Each digest was labeled with a fluorophore 2-aminobenzamide (2AB), and the 2AB-derivatives were separated by anion-exchange HPLC on an amine-bound silica PA-G column using a linear gradient of NaH_2PO_4, as indicated by the dashed line. The elution positions of 2AB-labeled CS/DS disaccharide standards in chromatograms of anion-exchange HPLC are indicated by numbered arrows: 1, ΔHexA-GalNAc; 2, ΔHexA-GalNAc(6S); 3, ΔHexA-GalNAc(4S); 4, ΔHexA(2S)-GalNAc(6S); 5, ΔHexA(2S)-GalNAc(4S); 6, ΔHexA-GalNAc(4S,6S); 7, ΔHexA(2S)-GalNAc(4S,6S).

Abbreviations: ΔHexA, GalNAc, 2S, 4S, 6S represent 4,5-unsaturated hexuronic acid, *N*-acetylgalactosamine, 2-*O*-sulfate, 4-*O*-sulfate, 6-*O*-sulfate, respectively.

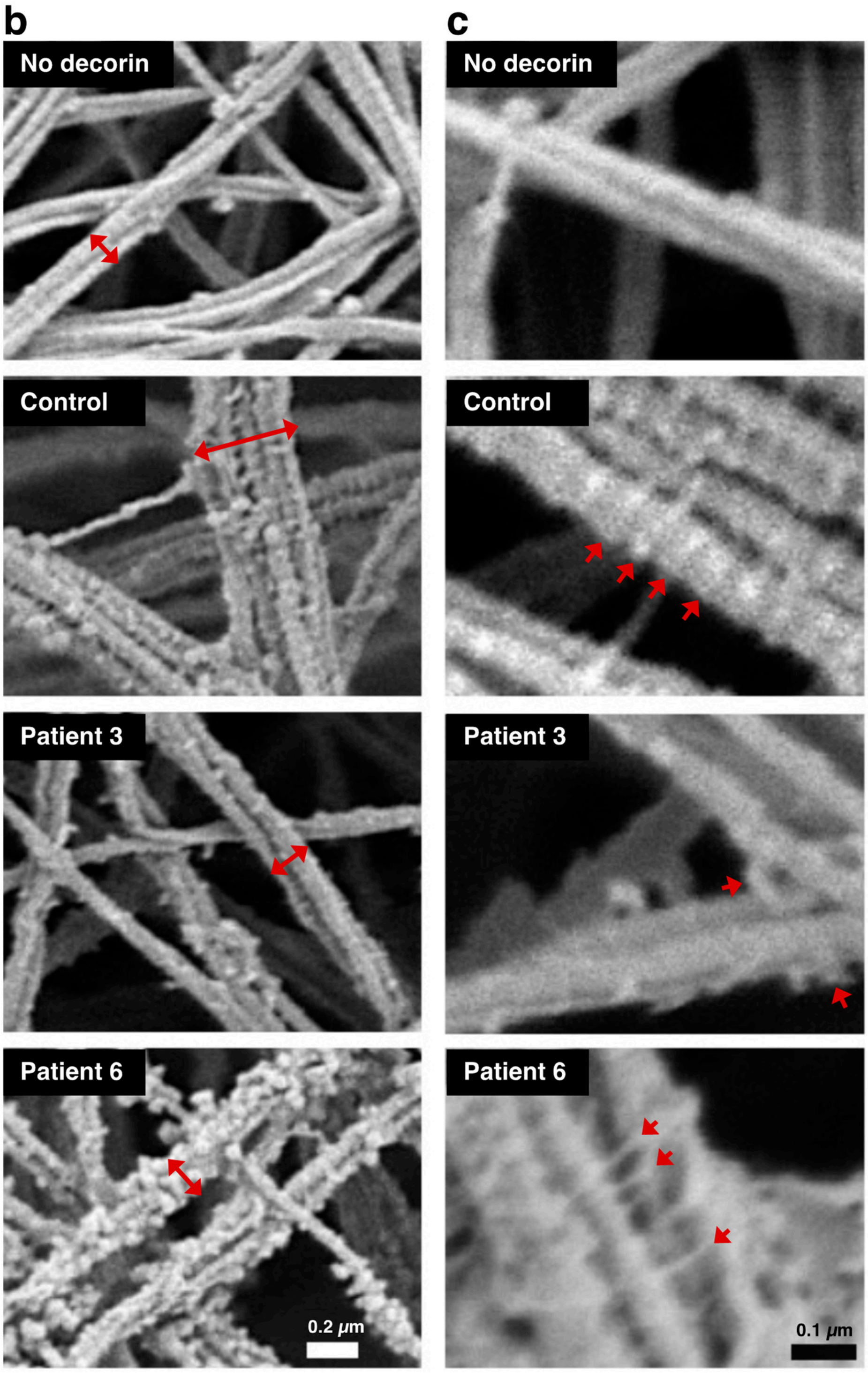

Supplemental Figure S2. The magnified images of Figure 4b and 4c. (b) Scanning electron microscopy analysis of collagen fibers incubated with or without decorin. The red two-headed arrow shows collagen fibers composed of multiple fibrils. **(c)** Observation at higher magnification visualized GAG chains as indicated by the red arrows.

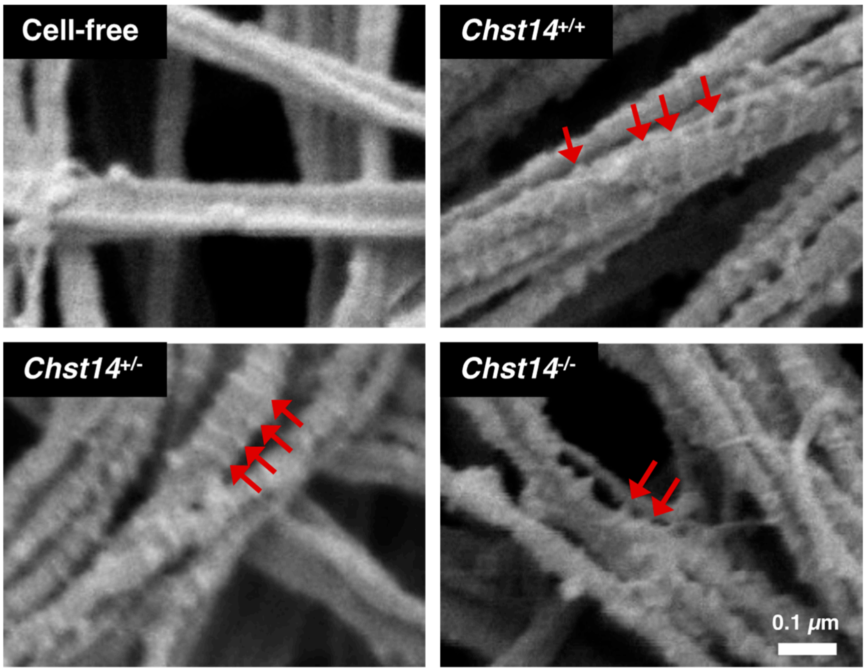

Supplemental Figure S3. The magnified images of Figure 5b. Scanning electron microscopy analysis of fibrils in the collagen gels co-cultured with or without fibroblasts from *Chst14*$^{+/+}$, *Chst14*$^{+/–}$, and *Chst14*$^{–/–}$ mice. The red arrows indicate GAG chains.
